# Microbial mats in the Turks and Caicos Islands reveal diversity and evolution of phototrophy in the Chloroflexota order Aggregatilineales

**DOI:** 10.1186/s40793-020-00357-8

**Published:** 2020-04-16

**Authors:** Lewis M. Ward, Usha F. Lingappa, John P. Grotzinger, Woodward W. Fischer

**Affiliations:** 1 Department of Earth & Planetary Sciences, Harvard University, Cambridge, MA 02138 USA; 2 Division of Geological & Planetary Sciences, California Institute of Technology, Pasadena, CA 91125 USA

## Abstract

Genome-resolved metagenomic sequencing approaches have led to a substantial increase in the recognized diversity of microorganisms; this included the discovery of novel metabolic pathways in previously recognized clades, and has enabled a more accurate determination of the extant distribution of key metabolisms and how they evolved over Earth history. Here, we present metagenome-assembled genomes of members of the Chloroflexota (formerly Chloroflexi or Green Nonsulfur Bacteria) order Aggregatilineales (formerly SBR1031 or Thermofonsia) discovered from sequencing of thick and expansive microbial mats present in an intertidal lagoon on Little Ambergris Cay in the Turks and Caicos Islands. These taxa included multiple new lineages of Type 2 reaction center-containing phototrophs that were not closely related to previously described phototrophic Chloroflexota—revealing a rich and intricate history of horizontal gene transfer and the evolution of phototrophy and other core metabolic pathways within this widespread phylum.

## Background

Most of the known diversity of phototrophic Chloroflexota (formerly Chloroflexi or Green Nonsulfur Bacteria) was derived from isolation- and sequencing-based efforts primarily on hot spring microbial mats (e.g. [35, 42]). However microbial communities with diverse members of phototrophic Chloroflexota are commonly found in other environments, including coastal marine environments and carbonate platforms (e.g. [36]). While cultivation-based efforts have yet to isolate phototrophic Chloroflexota from outside of the Chloroflexia class in pure culture [35], genome-resolved metagenomic sequencing has made substantial progress in uncovering novel diversity of phototrophic lineages that have otherwise remained inaccessible and unknown (e.g. [6, 19, 42, 48]).

**Fig. 1:**
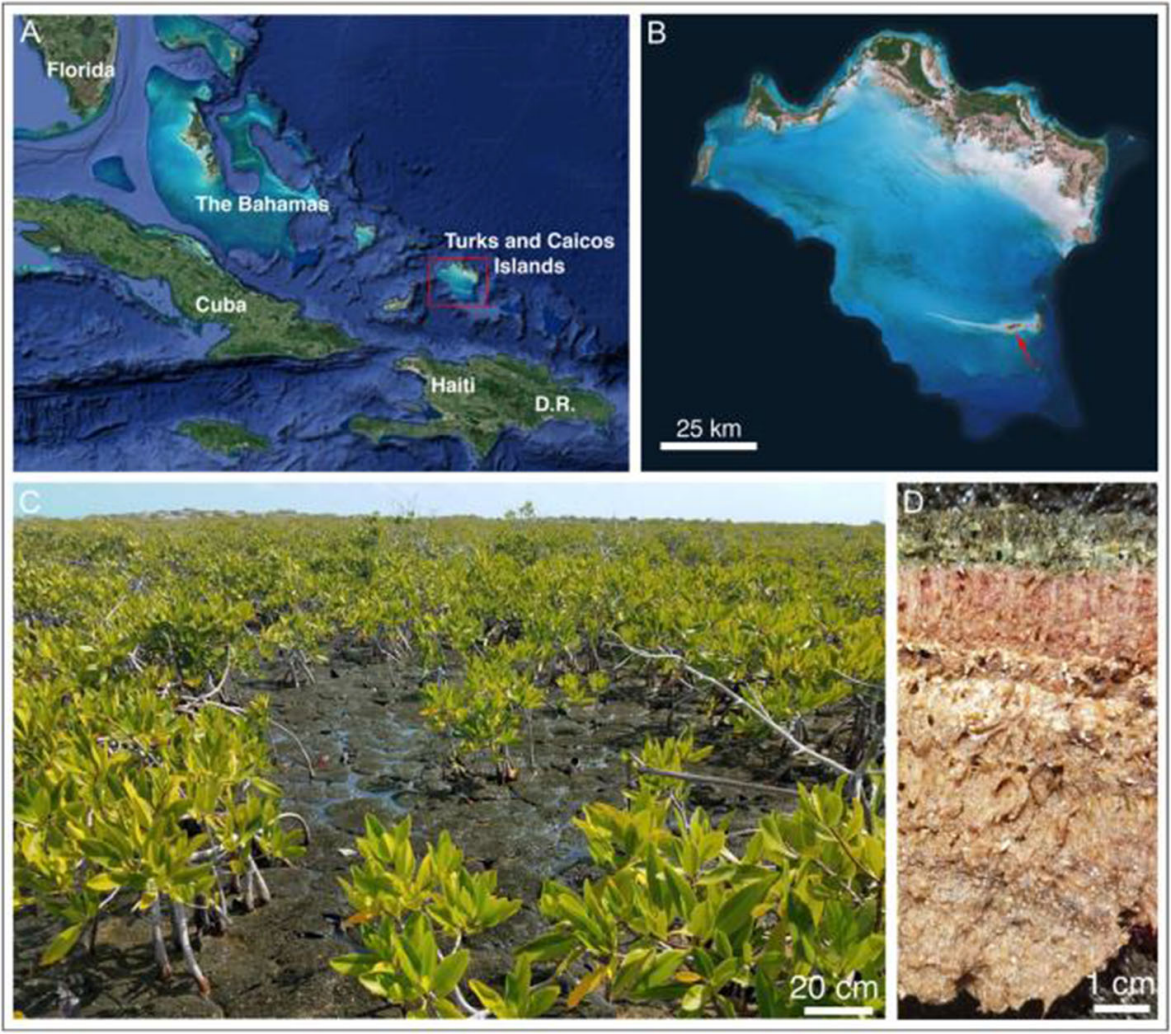
Geological context of the microbial mats from which the genomes in this study were recovered. **a** location of the Turks and Caicos Islands. **b** location of Little Ambergris Cay. **c** microbial mats and mangroves in tidal flat. **d** cross section of microbial mat showing pigmented layers containing phototrophic bacteria

The Turks and Caicos Islands occur at the southern end of the Bahamian archipelago. The Caicos platform (Fig. 1) is a contiguous shallow (< 5 m), grainy carbonate platform situated in the trade winds (mean wind velocity of 8 m/s from the east), and has a dry climate with net evaporation in excess of precipitation [10]. We studied microbial mats found throughout Little Ambergris Cay—a small ~ 6 km-long uninhabited island near the southern margin of the platform. Little Ambergris Cay contains a bedrock rim, formed by amalgamated cemented beach ridges and fossil eolian dunes, enclosing a tidal lagoon with a dozen large cut channels that communicate between the lagoon with well-mixed platformal waters. The typical diurnal tidal range is ~ 0.3 m. Polygonal microbial mats occur within the lagoon amongst sparse black mangroves. These mats are inundated daily by high tides. The mats are dissected into individual decimeter-sized heads that take on an upward domed shape by mature polygons with 60° angles created by episodic desiccation. On average twice a decade, tropical storms transport ooid sediment forming across the platform into the lagoon and onto the mats [37]—creating a mode of lamination in addition to that created by microbial succession. Dominant mat building taxa are a thickly-sheathed, heterocystous cyanobacterium of the genus *Scytonema* and the thin-walled cyanobacterium, *Halomicronema* [36]. The mats host a rich sulfur cycling community (Gomes et al.: Microbial mats on Little Ambergris Cay, Turks and Caicos Islands: taphonomy and the selective preservation of biosignatures/submitted). Deeper layers of the mat contain abundant purple/red- and green- colored microbes visible in exposed cross sections of the mats (Fig. 1); these have been confirmed by 16S rRNA gene amplicon sequencing to include diverse and abundant phototrophs in the Proteobacteria and Chloroflexota phyla [36].

In order to better characterize the diversity and evolutionary histories of phototrophic Chloroflexota, we recovered five metagenome-assembled genomes (MAGs) from genome-resolved metagenomic sequencing of microbial mats from the Turks and Caicos Islands which include novel phototrophic lineages of Chloroflexota not closely related to previously described phototrophs.

## Methods

Methods for metagenomic sequencing and genome binning followed those published previously [45, 46] and described briefly here. Samples of microbial mat were collected using an ethanol-sterilized spatula (~ 0.25 cm^3^ of material per sample). Immediately after sampling, cells were lysed and DNA preserved with a Zymo Terralyzer BashingBead Matrix and Xpedition Lysis Buffer. Lysis was achieved by attaching tubes to the blade of a cordless reciprocating saw (Black & Decker, Towson, MD) and operating for 1 min. Following return to the lab, bulk environmental DNA was extracted and purified with a Zymo Soil/Fecal DNA extraction kit. Purified DNA was submitted to SeqMatic LLC (Fremont, CA) for library preparation and sequencing via Illumina NextSeq.

Raw sequence reads from four samples were co-assembled with MegaHit v. 1.02 [22] and genome bins constructed based on nucleotide composition and differential coverage using MetaBAT [18], MaxBin [50], and CONCOCT [1] prior to dereplication and refinement with DAS Tool [32] to produce the final bin set. Annotation was performed using RAST [2]. Genome completeness and redundancy/contamination was estimated with CheckM [28], and likelihood of presence or absence of metabolic pathways was estimated with MetaPOAP [43]. Visualization of the presence of annotated metabolic pathways was done via KEGG-decoder [13] following annotation of proteins sequences by GhostKOALA [17]. Taxonomic assignments were verified with GTDB-Tk [8, 29].

Protein sequences used in analyses (see below) were identified locally with the *tblastn* function of BLAST + [7], aligned with MUSCLE [11], and manually curated in Jalview [49]. Positive BLAST hits were considered to be full length (e.g. > 90% the shortest reference sequence from an isolate genome) with *e*-values better than 1e^− 20^. Phylogenetic trees were calculated using RAxML [33] on the Cipres science gateway [25]. Transfer bootstrap support values were calculated by BOOSTER [20], and trees were visualized with the Interactive Tree of Life viewer [21]. Concatenated ribosomal protein alignments were built following methods from Hug et al. [16]. Evolutionary histories of vertical versus horizontal inheritance of metabolic genes were inferred by comparison of the topologies of organismal and metabolic protein phylogenies [9, 42, 47, 48].

## Results

**Fig. 2:**
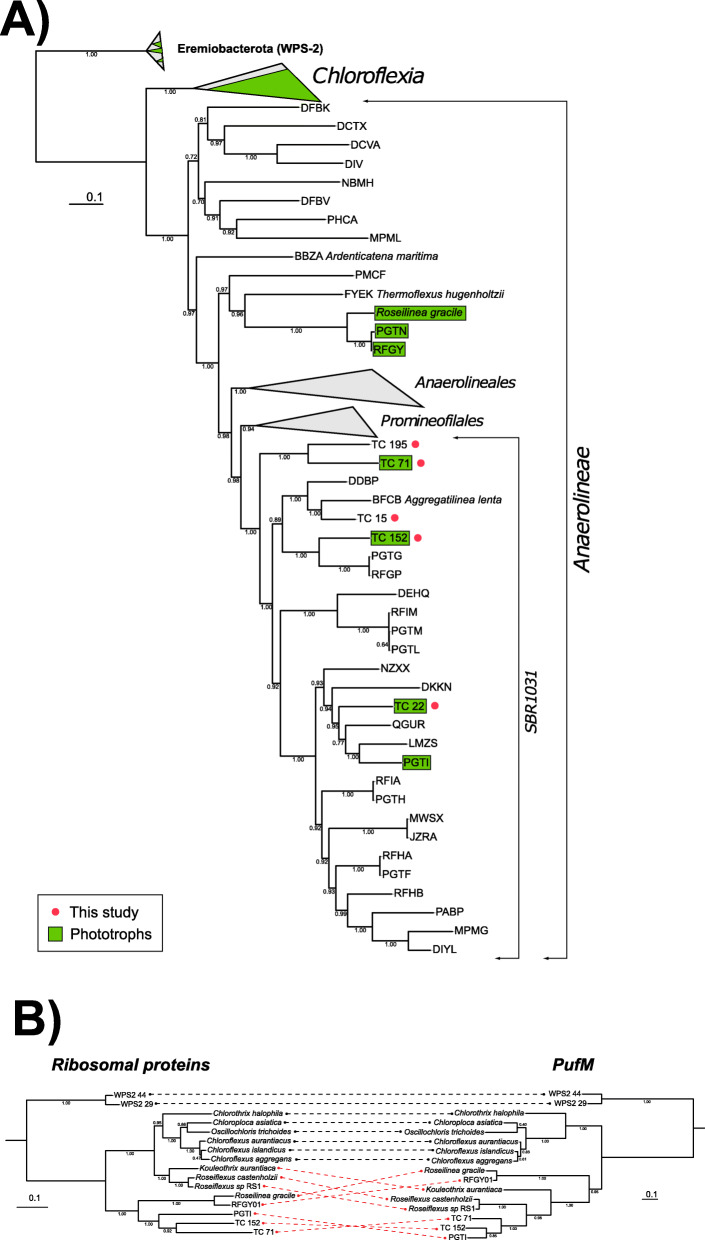
**a** Concatenated ribosomal protein phylogeny of the Chloroflexota, focusing on order SBR1031 (Aggregatilineales). Organisms encoding phototrophy with a Type 2 reaction center highlighted in green (including TC_22, which did not recover reaction center genes but may be a phototroph, as discussed in the text). Genomes first described here noted with pink circles. As species names are not available for MAGs of uncultured organisms, strains are labelled with MAG IDs (this study) or NCBI WGS database IDs (others) followed by taxonomy as derived from GTDB-Tk. Clades not the focus of this study have been collapsed and labeled with GTDB-Tk taxonomy. **b** Tanglegram showing phylogenetic (in)congruence between concatenated ribosomal proteins (left) reflecting organismal relationships with PufM (right) as a marker of the horizontal gene transfer of phototrophy proteins. Dotted lines show topological congruence within some lineages of Chloroflexia (in black) and incongruence between *Roseiflexus, Roseilinea,* and phototrophic members of SBR1031 (in red). This is indicative of horizontal gene transfer of phototrophy proteins from the *Roseiflexus* lineage to *Roseilinea* and SBR1031

Illumina NextSeq sequencing of four samples from the Turks and Caicos Islands produced a total of 243,782,642 reads of 151 nucleotides. These were coassembled into 3,464,316 contigs totaling 2,510,159,592 nucleotides. Binning of this dataset produced five medium- to high-quality genomes (according to accepted quality standards, [5]) which could be taxonomically classified by GTDB-Tk into the lineage of the Chloroflexota phylum currently annotated as order SBR1031 (Table 1, Fig. 2). Other genomes previously assigned to this clade included those proposed as the class *Candidatus* Thermofonsia [42] along with an isolate for which the order Aggregatilineales was recently proposed [26]. Following the isolation and characterization of *Aggregatilinea lenta* [26], and the clustering of these organisms into an order-level clade by GTDB-Tk, we propose the reassignment of the organisms previously described as “*Candidatus* Thermofonsia” into the order Aggregatilineales. This order is primarily made up of nonphototrophic organisms but also contains some members with full suites of phototrophy genes, which appear to have been derived via horizontal gene transfer from members of the Chloroflexia class of Chloroflexota [42].

**Table 1:** SBR1031/Aggregatilineales MAGs and genome statistics described in this study

Bin Id	Classification	Completeness	Contamination	Strain heterogeneity	Genome size (mb)	Coding sequences	GC%	Contigs	Shortest contig (nt)	RNAs	N50	Phototrophy markers (of 5)	Phototrophy False Positive estimate	Phototrophy False Negative estimate	NCBI WGS ID
TC_15	d__Bacteria;p__Chloroflexota;c__Anaerolineae;o__SBR1031;f__UBA2029;g__;s__	88.07	2.94	0	5.45	5127	60.1	662	2501	43	10,293	0	N/A	2.40x10^-5^	JAADYV
TC_152	d__Bacteria;p__Chloroflexota;c__Anaerolineae;o__SBR1031;f__;g__;s__	92.66	2.75	0	3.86	3445	55.7	312	2554	40	17,396	5	0.0372	N/A	JAADYW
TC_195	d__Bacteria;p__Chloroflexota;c__Anaerolineae;o__SBR1031;f__;g__;s__	95.41	0	0	4.06	4178	61.5	431	2518	44	11,646	0	N/A	1.98x10^-7^	JAADYX
TC_22	d__Bacteria;p__Chloroflexota;c__Anaerolineae;o__SBR1031;f__A4b;g__;s__	77.88	1.83	0	4.43	4167	61.7	746	2501	30	6808	3	0.0424	0.049	JAADYY
TC_71	d__Bacteria;p__Chloroflexota;c__Anaerolineae;o__;f__;g__;s__	87.61	1.33	33.33	4.06	4216	56.9	576	2481	44	8059	5	0.039	N/A	JAADYZ

## Discussion

The environmental range exhibited by known members of the SBR1031/Aggregatilineales was previously somewhat limited. Previously recovered genomes of members of this group were sourced from hot spring environments [42, 46], with the single known isolate isolated from subseafloor sediments [26]. 16S rRNA sequences of SBR1031 have been recovered from more diverse environments including hot springs [39, 40], contaminated soils [24], and wastewater [3]. Recovery of MAGs belonging to this order from carbonate tidal flats therefore expands the available genomic diversity and known range of Aggregatilineales to environments that also have an extensive geological record.

**Fig. 3:**
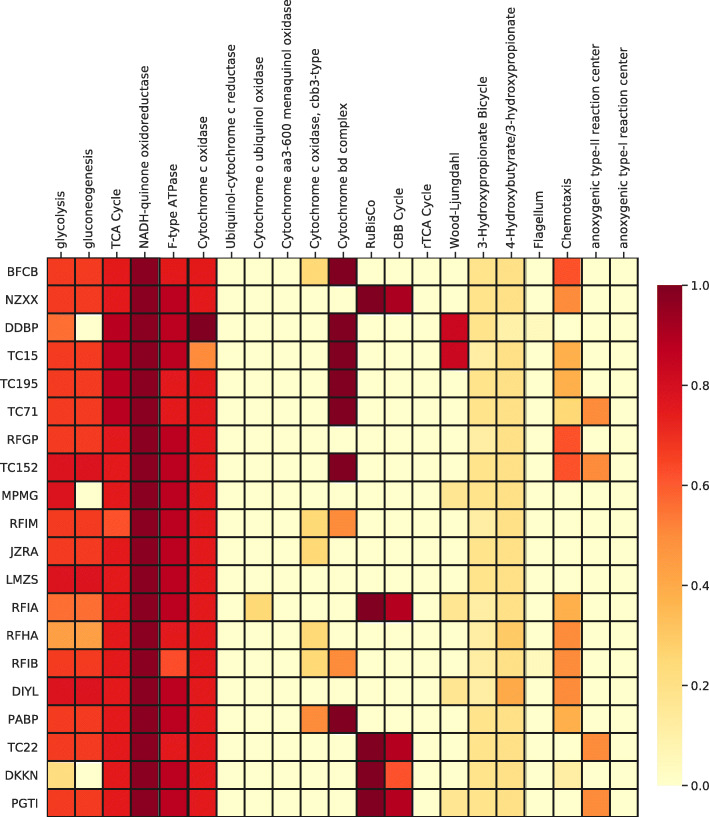
Heatmap of metabolic functions of Aggregatilineales genomes produced by KEGG-decoder. The color gradient reflects the fractional abundance of genes associated with a pathway encoded by a particular genome (i.e. white encodes 0 genes, and darkest red encodes all genes annotated as part of the pathway). Genome IDs as used in this study (TC##) or as WGS identifiers (all others). Note that the apparent presence of Calvin cycle genes in TC_22 and other members of Aggregatilineales is due to the presence of a Form IV rubisco-like protein that does not catalyze CO_2_ fixation, as described in the text

The SBR1031 MAGs reported here encoded similar sets of functional genes to previously reported members of this order (Fig. 3). Like previously described members of SBR1031/Aggegatilineales (e.g. [42]), these organisms encode aerobic respiration via an A-family heme copper O_2_ reductase, and contain both a *bc* complex and an alternative complex III [42]; based on these electron transport chain complexes, it is likely that these organisms are at least facultatively aerobic. All genomes described here (except for TC_22, the least complete genome) also encode a *bd* oxidase (O_2_ reductase) capable of functioning for O_2_ detoxification or respiration at low O_2_ concentrations [4]—a trait observed in both aerobic and anaerobic members of the Anaerolineae class (e.g. [14, 27, 38, 42]).

Of the five SBR1031/Aggregatilineales genomes reported here, three encode partial or full components necessary for phototrophic energy transduction via a Type 2 reaction center. TC_71 and TC_152 encode complete sets of marker genes for phototrophy, including those encoding PufL and PufM subunits of the reaction center and bacteriochlorophyll synthesis (e.g. BchX, BchY, and BchZ). MetaPOAP False Positive estimates for phototrophy in these organisms were low (< 0.04), suggesting that it is very unlikely that these genes were recovered as a result of contamination in the MAGs. The TC_22 genome encodes the BchXYZ complex but did not recover genes for PufL or PufM; MetaPOAP False Positive and False Negative estimates were similarly low (~ 0.04) for this genome, and based on this analysis it remains unclear whether or not this organism contains a complete set of genes for phototrophy. However, the gene cluster encoding BchX, BchY, and BchZ in TC_22 is located on the end of a contig, and the region of the chromosome syntenous to that encoding PufL and PufM in other phototrophic Chloroflexota (e.g. 6.5 kb upstream of *bchX, bchY,* and *bchZ* in TC_152) is missing in the TC_22 genome. Based on this, it is possible that this organism hosts a complete phototrophy pathway but that some genes simply were not recovered in the MAG. Like previously described phototrophs in SBR1031, these organisms do not encode a BchLNB complex or the capacity for carbon fixation via either the 3-hydroxypropionate bi-cycle or the Calvin cycle [42]. It is worth noting, however, that TC_22 does encode a Form IV rubisco-like protein on a small contig; however enzymes in this family are not capable of catalyzing CO_2_ fixation and instead are used for a variety of other functions [34] and this genome does not encode phosphoribulose kinase.

Comparisons of organismal (based on concatenated ribosomal proteins and other vertically inherited markers) and phototrophy protein (e.g. PufL, PufM) phylogenies indicated substantial incongruences between organismal and phototrophy tree topologies. These relationships are indicative of a history of horizontal gene transfer (Fig. 2b) (e.g. [30]). In particular, the reaction centers found in members of SBR1031 branch with those of *Roseiflexus* rather than as a clade separate from those of Chloroflexia (e.g. *Chloroflexus + Roseiflexus*), suggesting that horizontal transfer of phototrophy proteins occurred from the *Roseiflexus* branch to members of SBR1031—a pattern previously recognized in other members of the SBR1031/Aggegatilineales [42]. Interestingly, like *Roseiflexus* and some other phototrophic members of SBR1031/Aggegatilineales [42], TC_71 and TC_152 encode fused *pufL/pufM* genes encoding the two subunits of the Type 2 reaction center heterodimer. Together with reaction center protein phylogenic relationships, this observation indicated that the reaction centers of these organisms are more closely related to those of the *Roseiflexus* lineage of Chloroflexia than to the more closely related *Ca.* Roseilinea gracile, which encodes unfused *pufL* and *pufM* genes. A corollary of these observations of is that phototrophy in SBR1031 must postdate the acquisition and diversification of phototrophy in the Chloroflexia, events that have been estimated to have occurred in the last ~ 1 billion years [31].

## Conclusions

While horizontal gene transfer can be confidently determined to have been responsible for the presence of phototrophy in SBR1031, it remains unclear to what extent intra-order horizontal gene transfer played a role in the extant distribution of phototrophy within the clade. Phototrophic lineages of SBR1031 show a polyphyletic distribution, separated by many nonphototrophic lineages (Fig. 2a). This distribution could be reasonably explained by the presence of phototrophy in the last common ancestor of SBR1031 followed by extensive loss in most lineages; however differences in the topology of phototrophy proteins and organismal phylogenies within SBR1031 may indicate later acquisition followed by multiple instances of horizontal gene transfer between members of the clade. Discovery and study of additional phototrophic members of SBR1031 will be valuable to confidently resolve phylogenetic relationships of SBR1031 phototrophy proteins to assess organismal relationships in this clade.

The expanded environmental distribution and genetic diversity of Chloroflexota phototrophs described here further reinforces that genome-resolved metagenomic sequencing can provide an effective avenue for discovering novel microbial diversity, particularly of hard-to-culture phototrophs (e.g. [6, 15, 35, 41, 48]). These data also reinforce hypotheses that horizontal gene transfer has been a major mechanism behind the extant distribution of anoxygenic phototrophy (e.g. [12, 42, 48]). Together with the mounting evidence that most microbial lineages that have ever lived are now extinct [23], the continuing lack of discovery of donor lineages for horizontal gene transfer of phototrophy leads toward a consistent hypothesis: most phototrophic lineages that have ever existed have gone extinct, but relatively frequent horizontal gene transfer has allowed phototrophy pathways to persist in new lineages (e.g. [44]).

## Data Availability

The datasets generated during and analysed during the current study are available in the NCBI WGS repository under project ID PRJNA602167 with Accession IDs of JAADYW-JAADYZ.
